# One report, multiple aims: orthopedic surgeons vary how they use patient-reported outcomes with patients

**DOI:** 10.1007/s11136-022-03251-7

**Published:** 2022-09-14

**Authors:** Danielle C. Lavallee, Nan E. Rothrock, Antonia F. Chen, Patricia D. Franklin

**Affiliations:** 1 Michael Smith Health Research BC, Vancouver, BC Canada; 2 Feinberg School of Medicine of Northwestern University, Northwestern University, Chicago, IL USA; 3 Brigham and Women’s Hospital, Harvard Medical School, Boston, MA USA; 4 School of Population and Public Health, University of British Columbia, Vancouver, BC Canada

**Keywords:** Patient-reported outcomes, Shared decision-making, Total joint replacement

## Abstract

**Purpose:**

We conducted semi-structured qualitative interviews with surgeons to assess their goals for incorporating a patient-reported outcome measure (PROM)-based shared decision report into discussions around surgical and non-surgical treatment options for osteoarthritis of the knee and hip.

**Methods:**

Surgeons actively enrolling patients into a study incorporating a standardized PROM-based shared decision report were invited to participate in a semi-structured interview lasting 30 min. Open-ended questions explored how the surgeon used report content, features that were helpful, confusing, or could be improved, and how use of the report fit into the surgeon’s workflow. We used a conventional content analysis approach.

**Results:**

Of the 16 eligible surgeons, 11 agreed to participate with 9 completing the interview and 2 withdrawing due to work demands. We identified 8 themes related to PROM-based report use: Acceptability, Patient Characteristics, Communication Goals, Useful Content, Not Useful Content, Challenges, Training Needs, and Recommended Improvements. Additional sub-themes emerged for Communication Goals (7) and Challenges (8). All surgeons shared positive feedback about using the report as part of clinical care. Whereas surgeons described the use of the report to achieve different goals, the most common uses related to setting expectations for post-surgical outcomes (89%) and educating patients (100%).

**Conclusion:**

Surgeons tailor their use of a PROM-based report with individual patients to achieve a range of aims. This study suggests multiple opportunities to further our understanding of the ways PROMs can be used in clinical practice. The way PROM information is visually displayed and multi-component reports are assembled can facilitate diverse aims.

**Supplementary Information:**

The online version contains supplementary material available at 10.1007/s11136-022-03251-7.

Helping patients optimize their health requires attention to more than clinical markers and measures as drivers for health care decision-making [1]. It is increasingly recognized that patient-reported outcome (PRO) data provide meaningful insights into an individual’s physical, emotional, and social well-being. This is particularly true for chronic musculoskeletal conditions like knee and hip osteoarthritis (OA) where progressive pain and functional loss drive patients to seek operative and non-operative healthcare to relieve symptoms and improve quality of life. Consequently, efforts to embed routine PRO measurement into standard care practices for OA continue to gain traction. In orthopedics, PRO measures (PROMs) are considered standard of care for assessing OA severity and treatment outcomes and are utilized in care quality metrics by payers and policy makers.

Implementation of PROMs within health systems is complex, involving behavioral, technological, and organizational components [2]. All of these components need to be considered in order to fully assess effectiveness and to support replication when relevant [3]. Implementation science posits that soliciting users’ perspectives of an intervention can facilitate understanding of the determinants of its adoption [4]. The use of PROMs by orthopedic surgeons treating patients with OA offers one opportunity to understand characteristics of a PROM intervention that align with users’ needs.

OA is the most common form of arthritis and is the result of cartilage breaking down within the joint. OA has no cure but has multiple treatment options. The first line of treatment includes physical therapy, weight loss and activity modification, followed by pain medications aimed to reduce pain and improve mobility and physical function [5]. When these therapies no longer sufficiently relieve arthritis pain and function is impaired, total joint replacement (TJR) surgery is the most common treatment [6]. However, no clear guidelines exist for how to use PROMs to aid patients and clinicians in determining if or when to undergo TJR. To address this gap, the Arthritis care through Shared Knowledge (ASK) study deploys a PROM-based shared decision report to guide arthritis patients and their surgeons in discussions around surgical and non-surgical treatment decisions [7]. Printed and digital copies of the report are prepared for the participating surgeon and patient immediately prior to a consultation visit. Although over 15,000 patients at 14 clinical sites completed the PROM and risk assessment to generate the ASK report, it is unclear *how* clinicians use the report with the patient and which report components were most valuable to support treatment decisions. Therefore, we conducted semi-structured interviews with surgeons to assess their goals for and reflections on their experiences with the report.

## Methods

### ASK study and report

The ASK study is a pragmatic, cluster randomized controlled trial (ClinicalTrials.gov identifier: NCT03102580; 2018–2023) evaluating decision quality using a shared decision report (see Supplemental Material) designed through iterative consultation and engagement with clinicians and patients [7]. The ASK study used group level randomization at the site (office) level. A total of 36 surgeons at 10 different orthopedic physician groups (or practices) participated across 14 different locations including included medical school-based practices (8), community-based arthroplasty specialty practices (2), and community-based general orthopedic practices (4).

The intervention arm receives a 3-page report including individual level PROM and risk factor data collected from patients prior to the clinic visit. The report uses hip and knee specific PROMs (HOOS12, KOOS12) [8, 9] as well as measures of global physical and emotional health (SF-36 Physical Component Summary (PCS), Mental Component Summary (MCS)) [10]. It also includes anticipated 12-month pain and function outcomes based upon a validated predictive analytic algorithm using norms from the national Function and Outcomes Research for Comparative Effectiveness in Total Joint Replacement (FORCE-TJR) registry [7]. Specifically, our team generated six prediction models for primary knee and hip patients respectively as well as prediction models for three outcomes at 12 months: KOOS or HOOS pain, KOOS or HOOS function/ADL; and SF-36 PCS. The candidate predictors included demographic factors (age, sex, race, ethnicity), medical comorbidities (BMI, modified Charlson Comorbidity Index), musculoskeletal comorbidities (moderate or severe pain in low back (Oswestry) or non-operative hips and knees), and baseline status for the relevant outcome (pain, function/ADL, or PCS). These candidate predictors were identified based on orthopedic and OA literature and prior models [11–13].

The final page includes information about non-operative treatment options drawn from the American Association of Orthopedic Surgeons guidelines [14, 15]. The usual care arm receives a brief report of PROM and risk factor data without the estimated pain and function outcomes and non-operative treatment information.

### Participants

Eligibility criteria included surgeons participating in the ASK study for at least 6 months and actively enrolling patients into the study. All received a 3-page printed report with descriptive and predictive PROM information for patients considering total knee or hip arthroplasty. Of the participating 24 surgeons, 16 met criteria. All were invited to participate in a 30-min interview via video call. Interviews were recorded with verbal consent from all participants.

### Protocol

We created a semi-structured interview guide that addressed each section of the report. Open-ended questions explored how the surgeon used report content, features that were helpful, confusing, or could be improved, and how report use fit into the surgeon’s workflow. At the conclusion of the interview, participants were asked about number of years in clinical practice, experience with PROs during residency training, and if they collected PROMs for other patient populations or procedures outside of the ASK study. Two investigators (DCL, NER) used the interview guide with one surgeon participant and then revised the guide to improve clarity. The investigators then independently interviewed the remaining 8 participants via video call. Interviews were recorded, transcribed, and de-identified.

### Data analysis

We used a conventional content analysis to analyze data [16]. Both interviewers read all transcripts and independently created initial codes. Together they discussed and reconciled codes to produce a final codebook and definitions. Each independently coded all transcripts, then together reviewed coded transcripts to reconcile discrepancies. Next, both interviewers reviewed all excerpts assigned a given code to confirm or revise assignment as well as categorize concepts. For two concepts, sub-themes were identified. Again, each interviewer assigned sub-themes independently, then reconciled discrepancies together. To increase accuracy of data interpretation, we presented preliminary results to ASK surgeons and staff via webinar. Their feedback was then used to refine data interpretation. We used Dedoose v9.0.18 (Los Angeles, CA) for data management.

## Results

Of the 16 eligible surgeons, 11 agreed to participate with 9 completing the interview and 2 withdrawing due to work demands. Surgeons varied in number of years in practice (Table 1) and represented 6 of 11 eligible orthopedic practices. Approximately half of surgeons reported experience with PROs as part of their clinical training. All reported using the report during the patient consult with five (56%) also viewing the report prior to seeing the patient.

**Table 1:** Surgeon participants

# of years in practice (post residency)		
	< 5 years	3
	5–10 years	3
	11–19 years	0
	> 19 years	3
Utilized/collected PROs during orthopedic residency		
	Yes	5
	No	4
Time spent reviewing report during visit (min)		
	1–5	5
	5–10	2
	Unknown/varies	2
When report was used/referenced		
	Before patient consult	5
	During patient consult	9
PROMs collected outside of ASK study (i.e., different measures, different patient populations)		
	Yes	7
	No	2

We identified 8 themes: Acceptability, Patient Characteristics, Communication Goals, Useful Content, Not Useful Content, Training Needs, Challenges, and Recommended Improvements (Table 2). Additional sub-themes emerged for Communication Goals (7) and Challenges (8).

**Table 2:** Interview themes

Theme	Definition	Illustrative Quote, Participant ID
Acceptability	Comments regarding the global acceptability, helpfulness, or satisfaction of the report overall. Includes surgeon’s acceptance or the surgeon’s view of patients’ acceptance of the report	“I think this is very, very important data, and it’s really wonderful to have visually. I think people are really helped by it.” E
Patient characteristics	Surgeon’s descriptions of subgroups of patients (e.g., inquisitive patients, visual learners) and how report use by surgeon or patient changes accordingly	“I use the report most commonly on…patients that need a little bit more information, they’re kind of skeptical to begin with.” C
Communication goals	Surgeon’s goals for sharing information from the report with a patient	“At that first visit, using the pain numbers and the function numbers to explain where they are in their disease process in relation to other patients with the same disease.” I
Report content and configuration	Specific components of the report referenced during discussion. Defined either as **useful content** (i.e., either aids in discussion or provides information in a manner that supports discussion with patients) or **not useful content** (i.e., specific component(s) of the report that is not used or considered irrelevant.)	“…the patients find [pain and function graphs] incredibly helpful because they know how much they hurt, they know what they can do or they can’t do, but they don’t know where they are in comparison to other patients. So I think that’s one of the things this [report] does.” I
		“So, I probably don’t use [likely need for inpatient care after surgery] very much.” B
Training needs	Recommended training strategies or approaches for surgeons to use report or PROMs more broadly	“I think helping the surgeons understand how this [report] can help them in addition to how it can help the patients is really what probably most new surgeons need to understand.” I
Challenges	Specific report content that may impede discussions, impede workflow, or is challenging to understand	“I don’t know exactly what that [PCS] means for some patients...[They are] coming in for knee pain, and the knee pain is not too bad but the overall physical function is really bad. Or the opposite. And then, what exactly does that mean?” F
Recommended improvements	Specific recommendation for improving report content (e.g., adding X-ray score)	“If we could put the A1C on here. Because it’s not just whether or not they have diabetes, but how well controlled it is.” I
Training needs	Recommended training strategies or approaches for surgeons to use report or PROMs more broadly	“I think helping the surgeons understand how this [report] can help them in addition to how it can help the patients is really what probably most new surgeons need to understand.” I

### Acceptability and patient characteristics

All surgeons shared positive feedback about using the report. They described it as providing an easy, quick synopsis of helpful information similar to a case presentation. Surgeons liked the quantification of pain and physical function (e.g., “Giving some objective numbers to the patient [sic] symptoms, I think, is very, very important.” I). They reported patients liked the report and felt it increased patients’ knowledge of and confidence in the treatment plan. Surgeons noted that the report may be particularly helpful for older adults, patients with higher health literacy or numeracy, patients who are overly optimistic about their treatment outcomes, and patients that are seeking information or highly engaged in their care. Surgeons indicated the report may be less helpful for patients who are skeptical, don’t use the word “pain” to describe their symptoms, have lower health literacy or numeracy, have high self-efficacy (as no additional support or information is needed), and when a patient has already made a treatment decision (e.g., “And some patients are like, well, I already knew I wanted surgery anyways, so this is not changing my mind or making an difference in terms of my decision or outcomes.” D).

### Communication goals

Surgeons described using the report to achieve seven different goals (Table 3). All surgeons used different components of the report to **educate patients**. Education included giving patients context for how their pain and function compared with other patients (e.g., “…using the pain numbers and the function numbers to explain where they are in their disease process in relation to other patients with the same disease.” I). Surgeons also used the report’s information about surgical risk factors to highlight their impact on predicted pain, function, and general physical health outcomes (e.g., “I really go over what their predicted score changes will be and show them what comorbidities are affecting them that we can’t change. So, like low back pain, or whatever.” A).

**Table 3:** Communication goals when using PROM-based report

Participant	# of Goals	Education	Expectation setting	Shared decisions	Support treatment plan	Report as source of data	Coach behavior change	Re-frame progress after surgery
A	3	Y	Y	Y	–	–	–	–
B	5	Y	Y	Y	–	Y	–	Y
C	4	Y	Y	–	Y	–	–	Y
D	4	Y	Y	Y	Y	–	–	–
E	3	Y	Y	–	Y	–	–	–
F	3	Y	Y	–	–	–	Y	–
G	6	Y	Y	Y	Y	Y	Y	–
H	4	Y	Y	–	–	Y	Y	–
I	4	Y	–	Y	–	Y	–	Y
% of Surgeons with Goal	–	100%	89%	56%	44%	44%	33%	33%

Most surgeons (89%) used the report’s figures predicting function and pain following TJR to **communicate expectations** for post-surgical outcomes. They emphasized that they expected improvement, but not perfection (e.g., “We’re setting expectations that they will improve. But trying to make those expectations for improvement realistic.” B; “You’d have a lot of pain relief. You’d have much improvement in your knee function. Notice that it’s not 100%. So, we’re not looking for 100% on a knee replacement, but we’re looking for very good.” E). As the report figures displayed predicted outcomes at 12 months, surgeons used this feature to highlight the expected timeline for improvement (e.g., “And I say, ‘Look, these models are based on 12 months because people take 12 months to really get a final result.” A.)

Education and expectation setting accounted for 60% of all communication goals excerpted, with the remaining excerpts describing five other goals. All surgeons used the report for at least one of these additional goals, but no two surgeons had an identical set of communication goals. Goals were not correlated with whether or not the report was used prior to the visit. Five surgeons (56%) used the report to support **shared decision-making** (e.g., “They don’t want to undergo surgery. And I go, ‘I totally recognize that. Let’s see if there’s any other modalities that we can do.’” D). Four surgeons (44%) used the report as an additional **support for the treatment plan**, both for surgery and for non-surgical interventions (e.g., “I’ll kind of use this [report] as one more piece of information, to kind of support the decision that now is not the time [for surgery], or they’re not a good candidate for a joint replacement.” C). Four surgeons used the report as a **source of information** (e.g., “I start talking to the patient based off what I’m seeing [in the report].” G). One surgeon noted that sometimes the report identifies pain and functional problems on the contralateral side that the patient didn’t mention during their discussion. Three surgeons described utilizing the report’s indication of the presence of risk factors for surgery (e.g., body mass index [BMI], smoking, mental health) to **coach patients for behavior change** (e.g., “If these are the factors that we can modify, then we need to modify it to get a better outcome.” H). They also noted that patients may be more forthright about these factors in a patient-completed assessment than in conversation with the surgeon (e.g., “[patients] may not say that they are smokers, or they have emotional health issues, or narcotics use, so sometimes as they keep answering questions, it comes out.” H). Surprisingly, although the report captures and displays a patient’s pain and function prior to surgery, three surgeons referenced the report following TJR to **re-frame progress after surgery**. Specifically, they highlight how today’s pain and function compares to the pre-surgical report noting the timeline for improvement is 12 months (e.g., “You were at a 19, and this is where your pain is now. You’re now at a 97. That shows an incredible improvement.” I).

### Report content and configuration

Surgeons identified components of the report that were helpful for specific communication aims (e.g., graphs of hip and knee pain used to educate patients; predicted outcomes post-surgery used for expectation setting). Each component of the “Factors Affecting Your Arthritis Joint and Joint Replacement Outcomes” (i.e., BMI, emotional health, narcotics use, low back pain, medical comorbidity index, age) was identified by at least one surgeon as useful. Surgeons also identified aspects of the report that they didn’t use, which was most commonly information on non-surgical treatment options.

The report configuration was identified as helpful. This included having information that compared a patient to other patients. They also liked being able to compare hip pain to knee pain and the left side to the right side rather than having the report only include the joint discussed at that visit. Second, they found the use of color to convey score meaning (i.e., green, yellow, red) helpful, particularly when displayed in distinct tiers versus color gradations (e.g., greenish-yellow). Surgeons reported that patients benefited from having information displayed graphically and that both patients and surgeons liked having a physical, printed report (e.g., “I have patients who love this. They love the feedback of it because it gives them one, something tangible that leaves the office, which I think is very useful sometimes.” D).

### Challenges

Surgeons identified 8 challenges in using the report in practice. PROM score interpretation was the most frequently identified challenge in terms of number of surgeons (*n* = 8) and number of comments (*n* = 22). Surgeons described difficulty interpreting “overall physical function” and “general physical health” scores (e.g., “I don’t know exactly what that [PCS score] means for some patients. Because they’ll have – you know, you’re coming in for knee pain, and the knee pain is not too bad but the overall physical function is really bad. Or the opposite. And then, what exactly does that mean?” F). Similarly, some noted difficulty interpreting a “joint function” score that was a summary of joints, not specific to the joint being discussed (e.g., “But a lot of patients have complaints that are very specific to one joint, so I think that the function and physical health is not quite as concrete that the patient can understand.” A). Another noted challenge included different scales (e.g., range 0–100 for pain with 100 as goal (KOOS12/HOOS12); and 20–80 with 50 as norm for overall physical function (SF-36)) and lack of clarity on whether scores represented percentiles (e.g., “What does that 19 mean? You know, does that mean the 19^th^ percentile out of 100 people similar to it? Is it just an asterisk score which is 19?” F). Although all graphs had indications of the direction of scores, it was not always intuitive (e.g., “It looks like pain is increasing, and I have to say, pain is improving…But normally, when you think of your pain getting better, on a 10-point scale, the number goes down.” D). Surgeons reported that patients asked about the specific meaning of PROM items included in the report (e.g., “Sometimes patients talk about walking on a flat surface. They talk about, ‘Well, walking how far?…ten feet or…five miles?’” I).

A second commonly noted challenge related to workflow in clinic. The parent study’s staff were responsible for ensuring patient assessments were completed in advance of seeing the surgeon. Two sites provided an in-office option for patients to complete the assessment if not completed prior to arrival at the surgeon office. However, without this pre-visit study support, as one surgeon said, “We need an army of people of volunteers to get this information from patients.” (H). One surgeon described limitations in not having longitudinal reports (e.g., “The next visit when they come back, I don’t have [the report] with me and we finished talking about it, so there’s no way for me to kind of check back on it and see if they’ve addressed these things or not.” H). When asked to reflect on expanding use of PROM assessment in practice, surgeons raised concerns about PROM assessment on all patients slowing down clinic workflow, stressing the need for patients to complete PROMs before coming into the clinic. However, this is difficult, as some patients decline (e.g., “There are some patients who refuse to fill out the tablet if they’re there, or some who never filled it out at home…Some people say no to that.” D). They also pointed out the burden of accessing a report for all patients (e.g., “It’s going to be a little complicated to try and keep pulling this form out for every patient because on an average, if I see maybe somewhere like 40 to maybe 50 on a bad day, it would be hard to pull this up.” H).

Four surgeons expressed concern about the burden on patients to complete PROMs. This included concerns for multiple requests for different goals (e.g., “They’re normally bombarded by a lot of things, they’re bombarded by PROMs, they’re bombarded by questionnaires and things like that before even coming into the office when I see them.” D) and having those requests be unclear (e.g., “I still had, over the past couple of weeks, two or three patients coming and asking me that, ‘Did you want me to participate in this?’” H).

Another challenge was limitations for tailoring report content to a particular patient. One surgeon commented that he may weigh the use of narcotics differently for a patient with sickle cell disease or HIV, but the lack of that specific comorbidity information in the report limited the ability to fully tailor the report to each patient. Additionally, when the report was generated for a patient whose presenting issue was not related to OA, predicting outcomes post-surgery is no longer useful or relevant (e.g., “I think the problem is that the data is designed for people with hip arthritis. When you capture people that don’t have hip arthritis, the data doesn’t work correctly.” C).

Other challenges included times when a patient was seen for one joint (i.e., left hip), but the contralateral side (i.e., right hip) indicated worse pain leading to concern about underlying reason for lack of concordance in report information and patient report. In addition, the lack of standardization in what PROMs are used in clinical care was a noted challenge for efficiency in data capture and ability for scalability. Surgeons expressed concerns about the expected increased mandates for capturing PROMs for billing requirements, and one described a divide within the field about whether or not PROMs are sufficiently useful to warrant implementation costs (e.g., “There are still leaders in the field that are arguing that it’s a complete waste of time.” I).

### Recommended improvements

Surgeons identified modifications to improve interpretability, such as modifying figure labels, increasing visibility of specific information, use of consistent PROM scales (e.g., all 0–100), and supporting printing in black/white. Other recommendations focused on adding additional medical information such as x-ray grade, diagnosis, and hemoglobin A1c. An interest existed for displaying the timeline for improvement (e.g., adding expected outcomes at 6 months) and creating longitudinal reports showing patient PROM scores over time. Finally, there were suggestions to expand information on non-surgical treatment options with predictive analytics indicating expected outcomes and duration of treatment impact.

### Training needs

Most surgeons (78%) had used PROMs in clinic prior to the study. However, when asked about training needs, surgeons suggested additional training beyond the study-provided standard, one-time training on the value of engaging patients in treatment decisions, as well as the content and use of the information in the report. Surgeons suggested training on how the report can help them, how to use it with a patient, and specific score interpretation guidance. They suggested training should be brief (e.g., 10–15 min) and utilize a case-based approach (e.g., trainer demonstrating report use with a hypothetical patient).

## Discussion

Inclusion of patient-reported pain and function data to guide discussions regarding treatment options is essential when such outcomes serve as primary goals of treatment, as is the case with knee and hip OA. Much of the PROM implementation literature focuses on logistics, patient engagement, and informatics for completion of measures. This is one of first papers to define how clinicians use PROMs in providing care for patients with OA when data are integrated in a standardized report.

Within the same patient population and same type of consultation, surgeons used PROM data to achieve different and multiple aims. Patients presenting for surgical consultation for OA are at different stages of disease progression and decision-making (e.g., early discussions about surgery versus seeking a second opinion). As a result, a range of visualizations of different types of information are warranted to support the range of goals of patients and surgeons in a clinical visit.

The multi-component ASK report (e.g., PROM data, risk factors, predicted outcomes) supports this variation in use. For example, information about how a patient’s pain and function compares with others can facilitate a shared understanding between a patient and surgeon. For patients seeking surgery, predicted pain and function post-TJR can facilitate a shared expectation for symptom and health improvement, but not perfection. Surgeons described tailoring what content they used to the individual patient’s status. This aligns with the construct of design quality from the Consolidated Framework for Implementation Research (CFIR) [4]. Bundling multiple interpretable components in a single report supported adapting use for a specific patient. However, including information that is not relevant for a given patient in a standard report may result in confusion for patients and challenges for surgeons. For example, when patients not considering surgery viewed predicted post-surgical outcomes, they raised questions and concerns. Balancing the generalizability of the content in a standardized report with content specifically tailored to an individual patient is needed. The extent to which this balance can be improved through dynamic report generation using information technology tools warrants further study.

Involvement of the end-users of data is a critical step in design. The ASK report was developed, reviewed, and tested by surgeons and patients throughout the design phase. This is reflected by the high acceptability of the report by surgeons, their perception of the relative advantage of the report, and their comfort with accurately interpreting PROM data across diverse use cases. Even so, participants identified additional opportunities to make the report more understandable and useful in clinical care. This included how information was displayed as well as what content was provided. For example, the inclusion of non-operative treatment options was recommended during the design. However, this study revealed use of this information was limited. Reasons included utilization of other educational resources or addressing non-operative options at times other than when reviewing the ASK report. This highlights the importance of iterative design processes as new users with real-world experience with a tool can identify opportunities for further refinement that support improved feasibility and accuracy. Enabling mechanisms for greater adaptability in report content that meets users’ needs may aid future adoption.

This study has several limitations. Participants in interviews represent a highly selective sample. Eligible surgeons opted in to a study requiring the integration and use of a PROM-based reports in their clinical practice, and therefore reflect a sample of surgeons interested in capturing and using such data in practice. In addition, not all surgeons agreed to participate in interviews and thus responses may reflect a more positive experience. Although the surgeons involved reflect diverse practice sites and years in practice, the small sample size limits the generalizability of findings, particularly surgeons in smaller practices. For example, 2 of the non-participating practices included only 1 eligible surgeon who declined to participate. While the interview number is small, we believe that reporting these themes is an important first contribution to the limited literature about how PROs are used in practice. Finally, interviews focused on the use and content of the report. It is important to note the study invested significant resources to facilitate office workflows that engage patients in PROM completion and put the printed report in front of surgeon prior to the patient visit. Workflow barriers for PROM integration are well documented [17–19] and not addressed in this analysis.

This study suggests multiple opportunities to further our understanding of the ways PROMs can be used in clinical practice. First, PROMs are increasingly integrated in the management of chronic conditions and in conditions where PROMs are not assessing the primary goals of care (e.g., early-stage cancer). It is important to conduct additional research to better understand how PROM use varies across contexts (e.g., acute versus chronic care, primary versus secondary outcomes). Furthermore, having access to validated, normative predictive algorithms for PROMs tailored to an individual patient is rare, but was some of the most heavily utilized information. The ways a single assessment or longitudinal assessment without predictive information alters the repertoire of aims for PROM utilization is unknown. Finally, research on the extent to which patients share goals for communication about PROMs and experience utilization of the ASK report is underway.

In conclusion, surgeons tailor their use of a standardized PROM-based report with individual patients to achieve a range of aims. Patient education and setting expectations were identified as the most common uses of PROMs in this setting. The challenges experienced (e.g., score interpretation, patient burden) are not specific to this report, and have been documented in other implementations [17–19]. There are opportunities for report refinement based on surgeons’ repeated use in practice. Surgeons may benefit from additional education focused on expanding their repertoire of ways to use PROMs for patient care. Knowing providers’ aims for PRO data use can inform future implementations of PROMs in clinical practice by helping to identify key components to include.

## Supplementary Information

Below is the link to the electronic supplementary material.

Supplementary file1 (PDF 179 kb)

## Abbreviations

ASK: Arthritis Shared Knowledge study
MCS: Mental Component Summary
OA: Osteoarthritis
PCS: Physical Component Summary
PRO: Patient-reported outcome
PROM: Patient-reported outcome measure
TJR: Total joint replacement
